# Single molecule resolution of the antimicrobial action of quantum dot-labeled sushi peptide on live bacteria

**DOI:** 10.1186/1741-7007-7-22

**Published:** 2009-05-11

**Authors:** Sebastian Leptihn, Jia Yi Har, Jianzhu Chen, Bow Ho, Thorsten Wohland, Jeak Ling Ding

**Affiliations:** 1 Singapore-MIT Alliance, 117576, Singapore; 2 Koch Institute for Integrative Cancer Research and Department of Biology, Massachusetts Institute of Technology, Cambridge, MA 02139, USA; 3 Department of Microbiology, Yong Loo Lin School of Medicine, National University of Singapore, 117597, Singapore; 4 Department of Chemistry, National University of Singapore, 117543, Singapore; 5 Department of Biological Sciences, National University of Singapore, 117543, Singapore

## Abstract

**Background:**

Antimicrobial peptides are found in all kingdoms of life. During the evolution of multicellular organisms, antimicrobial peptides were established as key elements of innate immunity. Most antimicrobial peptides are thought to work by disrupting the integrity of cell membranes, causing pathogen death. As antimicrobial peptides target the membrane structure, pathogens can only acquire resistance by a fundamental change in membrane composition. Hence, the evolution of pathogen resistance has been a slow process. Therefore antimicrobial peptides are valuable alternatives to classical antibiotics against which multiple drug-resistant bacteria have emerged. For potential therapeutic applications as antibiotics a thorough knowledge of their mechanism of action is essential. Despite the increasingly comprehensive understanding of the biochemical properties of these peptides, the actual mechanism by which antimicrobial peptides lyse microbes is controversial.

**Results:**

Here we investigate how Sushi 1, an antimicrobial peptide derived from the horseshoe crab (*Carcinoscorpius rotundicauda*), induces lysis of Gram-negative bacteria. To follow the entire process of antimicrobial action, we performed a variety of experiments including transmission electron microscopy and fluorescence correlation spectroscopy as well as single molecule tracking of quantum dot-labeled antimicrobial peptides on live bacteria. Since *in vitro *measurements do not necessarily correlate with the *in vivo *action of a peptide we developed a novel fluorescent live bacteria lysis assay. Using fully functional nanoparticle-labeled Sushi 1, we observed the process of antimicrobial action at the single-molecule level.

**Conclusion:**

Recently the hypothesis that many antimicrobial peptides act on internal targets to kill the bacterium has been discussed. Here, we demonstrate that the target sites of Sushi 1 are outer and inner membranes and are not cytosolic. Further, our findings suggest four successive steps of the bactericidal process: 1) Binding, mediated mainly by charged residues in the peptide; 2) Peptide association, as peptide concentration increases evidenced by a change in diffusive behavior; 3) Membrane disruption, during which lipopolysaccharide is not released; and 4) Lysis, by leakage of cytosolic content through large membrane defects.

## Background

The innate immune system provides the first line of defense against invading pathogens. Amongst various effectors, antimicrobial peptides (AMPs) which are found in all eukaryotes [1], are potent agents against a wide range of pathogens, including Gram-positive bacteria (GPB) and Gram-negative bacteria (GNB), fungi and protozoa [2]. AMPs range from 9 to 54 amino acid residues in length and are usually positively charged. Based on their structures, cationic AMPs are divided into four major classes: α-helical peptides, β-sheet peptides which are stabilized by up to three disulfide bridges, loop structures containing only one disulfide bridge, and extended structures with a predominance of one or more amino acids [3-7]. While β-sheet peptides are structured in solution even before interaction, peptides from the α-helical class exist as disordered structures in aqueous media. Some of these peptides, for example cecropins [8], magainins [9], and melittins [10], become amphipathic helices upon interaction with the hydrophobic membranes of bacteria.

A major component of the outer membrane of GNB is lipopolysaccharide (LPS). When released, LPS stimulates a strong inflammatory response in the host, which can lead to septic shock [11-13]. The Sushi 1 (or S1) peptide derived from Factor C of the horseshoe crab is extensively characterized [14-20]. It is an α-helical cationic AMP, which binds LPS with high affinity. The 34-amino acid S1 contains a motif with alternating hydrophobic and basic residues that are thought to be important for the interaction with LPS [21].

The mechanisms underlying the potent and rapid bactericidal activities of AMPs have been widely investigated [22-29]. According to the hypothesis of self-promoted uptake, the electrostatic attraction between the negatively charged LPS and the cationic peptide is important for the interaction of the peptides with the bacterial surface [30,31]. Cationic peptides have a higher affinity than do native divalent cations for membrane-embedded LPS and thereby destabilize the targeted areas, facilitating the translocation of the peptide through the outer membrane [32]. Once the peptide has crossed the outer membrane and the mesh-like peptidoglycan cell wall, it is envisaged to interact with the negatively charged surface of the cytoplasmic membrane. To explain the mechanism of antibacterial action of membrane-active AMPs, several models have been proposed [23,24,26,27,33,34]. According to these models, the mechanism of action is thought to be the breakdown of membrane integrity by inducing pores or by a detergent-like membrane disruption leading to leakage of the cytosolic content.

Although the lipid-binding and antimicrobial effects of AMPs have been intensively studied, the mechanisms by which AMPs kill bacteria remain controversial. Moreover, the dynamics of bactericidal processes are relatively unknown. To date, most of the experiments and simulations that have aimed to elucidate the bactericidal mechanisms have been carried out with artificial membranes but not with live bacteria. The drawback of this approach was demonstrated recently by observations that leakage experiments with artificial membranes are not a reliable indicator for the prediction of antimicrobial activity *in vivo *[35]. In addition, most experiments to date have been carried out on ensembles, which may mask heterogeneous behavioral characteristics of the individual AMP molecules.

Here we report for the first time the use of a combination of high-resolution imaging, *in vivo *single molecule observation, and biochemical and biological functional assays to investigate the mechanism of action of a fully functional nanoparticle-labeled antimicrobial peptide. After treatment of bacteria with nanogold-labeled S1 and fixation, we made endpoint observations of bacterial killing using transmission electron microscopy (TEM). Real-time measurements of the dynamics of quantum dot-labeled S1 on live bacteria were performed by fluorescence correlation spectroscopy (FCS) and single particle tracking (SPT). To exclude the potential unreliability of the antimicrobial lysis assays, we developed a live bacterial lysis assay using *Escherichia coli *that expresses a fluorescent protein. The combination of a high resolution single molecule imaging technique and *in vivo *bacterial lysis assays have allowed us to elucidate the mechanism of action of S1 with direct physiological relevance.

## Results

### Nanoparticle-labeled S1 retained its specific activity

**Figure 1:**
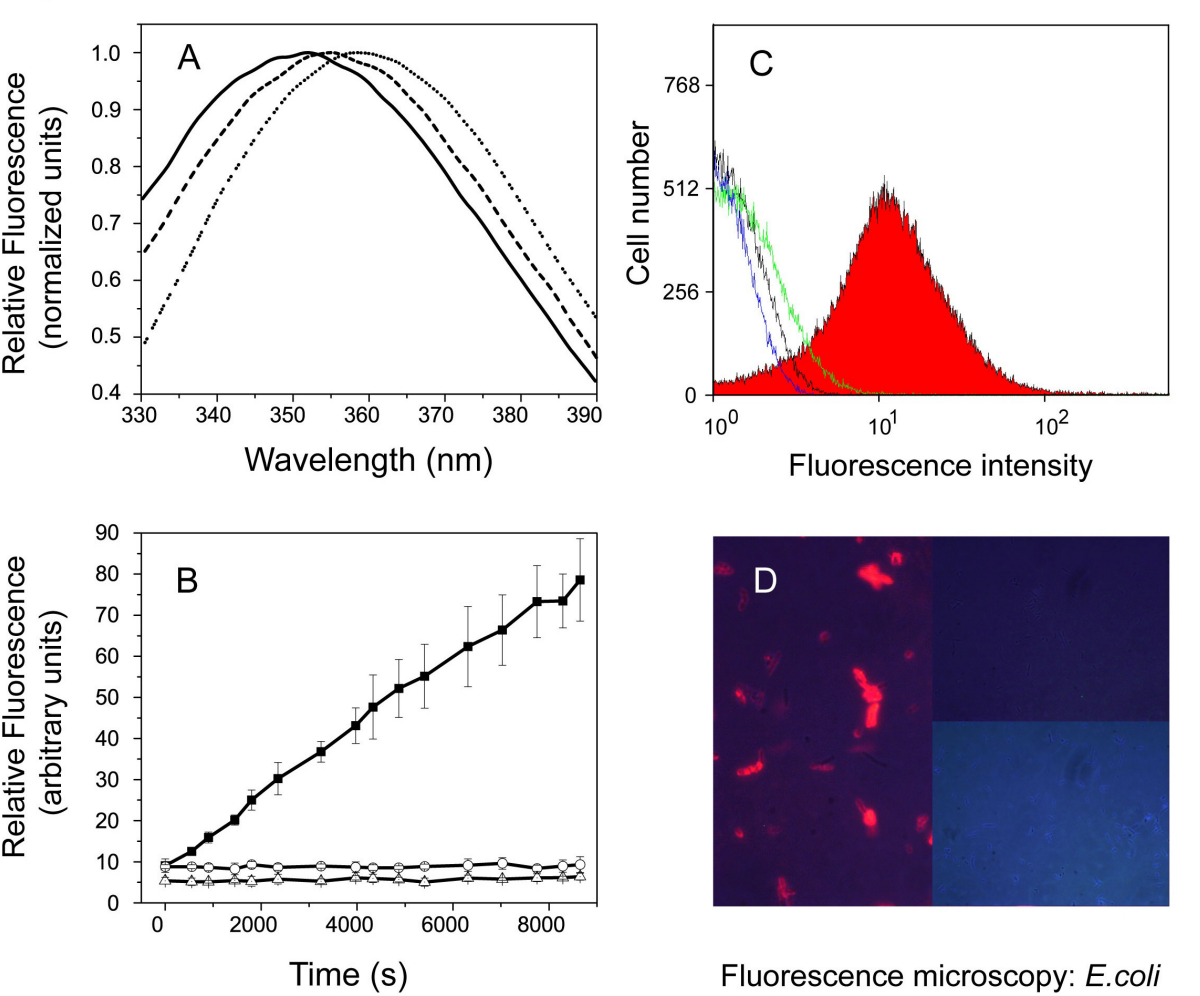
**Qdot-peptide conjugate probe testing**. (A) Tryptophan fluorescence emission spectra of S1 in buffer (dotted line), bound to Qdots (dashed line) or bound to Qdots in POPS (solid line). The samples contained either only 500 nM peptide or 500 nM Qdot-peptide conjugates with or without 25 μM POPS. (B) Lipopolysaccharide (LPS) neutralization assay. Recombinant Factor C activation assay of unlabeled S1 (open circle) or as a Qdot conjugate (open triangle). The activation of Factor C without LPS binding peptide is displayed with a solid square. The samples contained 125 nM S1 or S1-Qdot conjugate (1:1 ratio) or no peptide. Non-conjugated Qdot as well as negS1 showed no neutralization ability in this assay. (C) Flow cytometry histogram of Qdot-labeled *Escherichia coli*. Biotin-Qdot (black line) shows very little fluorescence similar to the *E. coli *auto-fluorescence (blue line). The Qdot-negS1 labeled *E. coli *cells show minor fluorescence (green line). The mean fluorescent intensity for Qdot-S1 labeled cells is clearly separated and indicates the specific staining of the cells by the probe (red). Settings: Excitation 488 nm, emission 675 nm, counted cells 100,000. (D) Staining of *E. coli *with Qdot-S1 conjugate. Overlay of light microscopy and fluorescence images. Intense staining is observed by using Qdots-S1 peptide (left). The controls with biotin (upper right) or negS1 (lower right) show that the unspecific binding was minimal. Total magnification: 400×.

To study S1 at a single-molecule level, we designed nanoparticle conjugates making use of the strong biotin-streptavidin interaction. We performed a variety of tests to be certain that conjugation of Qdot to S1 does not affect the peptide activity (Figure 1).

Firstly, the Qdot label did not interfere with S1 interaction with membrane phospholipids. The tryptophan residue in S1 exhibits a characteristic blue shift in emission when entering a non-polar environment. Unlabeled S1 showed a 10 nm shift in anionic phospholipids, such as POPS or POPG [36]. Similarly, the tryptophan residue in S1-Qdot showed the same spectral shift upon interaction with lipids such as POPS (Figure 1A), suggesting that S1-Qdot has retained its ability to insert into the non-polar environment of the lipid. The control negS1 showed no spectral shift in any of the lipids tested ((A) in Additional file 1) demonstrating the specificity of the interaction between S1 and the bacterial membrane lipids.

Secondly, Qdot-conjugated S1 neutralizes LPS as efficiently as unlabeled S1, thus showing identical biological activity. When activated by LPS, recombinant Factor C (rFC) cleaves a fluorogenic substrate. Neutralization of LPS by S1 prevents rFC from cleaving the substrate. At a concentration of less than 75 nM, S1-Qdot inhibited rFC to the same extent as S1 (Figure 1B), suggesting that Qdot labeling of S1 did not affect its binding and neutralization of LPS. The control, negS1, did not neutralize LPS (Additional file 1 (B)).

Thirdly, surface plasmon resonance experiments confirmed binding of S1-Qdot to lipid A, the bioactive part of LPS. The affinity of S1 and S1-Qdot towards lipid A was 9.3 × 10^-9 ^M and 6.1 × 10^-8 ^M, respectively. The binding is specific because biotin-Qdot conjugates did not bind to lipid A.

Finally, S1-Qdot specifically and strongly interacted with GNB. Following incubation of bacteria with S1-Qdot, the samples were extensively washed prior to Fluorescence-activated cell sorting analysis (Figure 1C). Bacteria were all positively stained with S1-Qdot. Interestingly, the fluorescence emission of S1-Qdot bound to the bacteria was blue-shifted by 22 nm to 533 nm (Additional file 1), similar to an observation reported for aptamer-conjugated Qdot [37]. In addition, when *E. coli *were incubated with S1-Qdot, they became highly fluorescent (Figure 1D). With negS1-Qdot, almost no signal could be obtained under the same conditions, similar to cells incubated with biotin-Qdot. Taken together, these results demonstrate that despite its size and chemical properties, the nanoparticle does not significantly affect the biochemical properties and functional behavior of the conjugated S1.

### S1-nanogold penetrates outer and inner membrane and enters the bacterial cytosol

**Figure 2:**
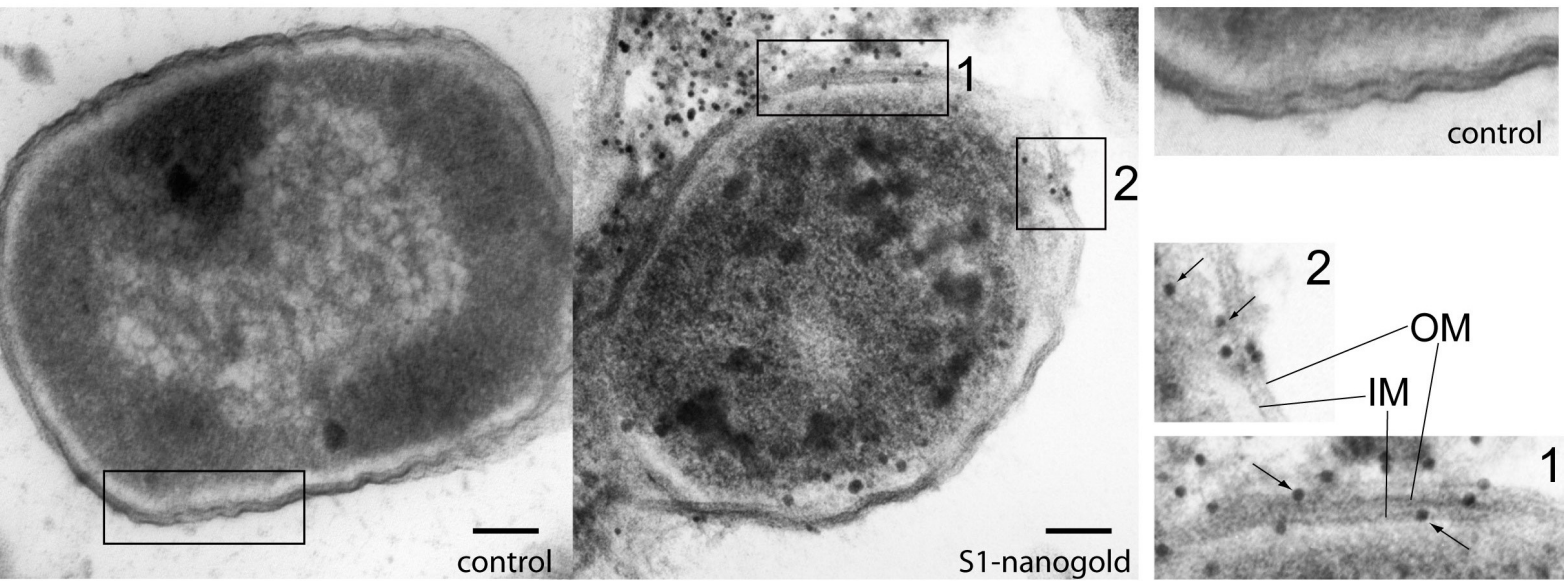
**Electron microscopy of S1 nanoparticles on *Escherichia coli***. Transmission electron microscopy micrograph of *E. coli *with negS1 (left) or S1-nanogold conjugate (middle). An ultrathin section of a cell incubated with 1 μM negS1- or S1-nanogold conjugate, fixed with paraformaldehyde, glutaraldehyde and osmium tetroxide. The right panel shows 2× magnified details. The 'control' shows a membrane section of negS1-nanogold, whereas (1) and (2) are details of S1-nanogold. Nanogold particles, identified as electron-dense black spots (arrows), can only be found in samples containing S1-nanogold particles and are distributed on the outer and inner leaflets of both membranes, as well as in the periplasm and cytoplasm. OM: Outer membrane, IM: Inner membrane. Scale bar: 100 nm.

The spatial distribution of the AMPs in and on the bacteria was determined by TEM using nanogold-conjugated S1 as a reporter of S1 localization. After removing unbound particles by washing, the reaction was stopped using reagents which cross-link peptides, proteins, carbohydrates, lipids and nucleic acids. The nanoparticles were found on the inner and outer leaflets of the inner and outer membranes, as well as in the periplasmic space and cytosol of the bacterium (Figure 2). Controls with nanogold alone confirm that this result is due to peptide functionality. We included negS1-nanogold as well as biotin-nanogold as controls. In these controls, particles could not be observed, neither free nor bound to bacteria. A further control included the use of both components separately (non-biotinylated S1, biotin-nanogold), not as a peptide-nanogold conjugate. Again, in all controls, particles could not be observed (*n *< 200). Therefore it can be concluded that only particles conjugated with S1 penetrate the membranes and enter the cell, demonstrating the specificity of the S1-nanogold.

To quantify particle distributions, we counted particles on or in the cells in TEM images (*n *= 254). When 1 μM S1 was used, approximately 77% of the particles were found attached to the outer leaflet of the outer membrane; less than 9% with the inner leaflet of the inner membrane; less than 7% in the cytosol; and 8% in the periplasmic space (Additional file 2). These results suggest that S1 binds and penetrates the outer and inner membranes to disrupt the lipid bilayers of both membranes, allowing particles up to 10 nm to diffuse through the membrane barrier.

### S1 induces immediate leakage of bacterial cytosolic content without release of lipopolysaccharide molecules

To determine the kinetics of bacterial killing by S1 in real time, we used FCS to measure leakage of green fluorescent protein (GFP) from GFP-expressing *E. coli*. FCS is based on fluorescence intensity fluctuations within a confocal volume. From these autocorrelation functions (ACF) can be determined which can be fitted with theoretical pre-determined models to extract molecular parameters for different fluorescence species [38]. In particular, by using FCS it is possible to determine the average time a particular fluorescent species takes to traverse the confocal volume and the number of particles within this confocal volume. The diffusion time (T_d_) is directly related to the diffusion coefficient (*D*) since in FCS the following equation holds for diffusive processes: *D*·T_d _= constant

**Figure 3:**
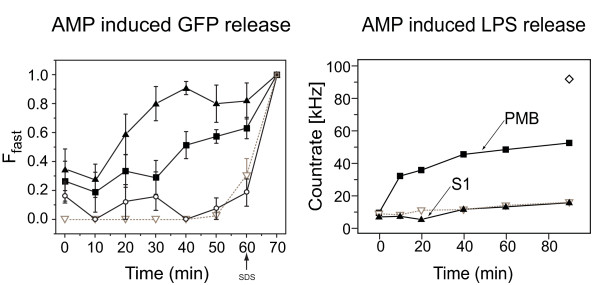
**Determination of green fluorescent protein release from *Escherichia coli *using fluorescence correlation spectroscopy**. (A) Bacterial lysis assay with 500 nM S1 (solid upward triangle), 500 nM polymixin B (PMB) (solid square), no additive (open downward triangle) and negS1 (open circles) using fluorescence correlation spectroscopy (FCS). The peptide-induced leakage of green fluorescent protein (GFP) from the cytosol was observed by recording the fluorescence correlation curves and fitting it after a two-particle diffusion model. F_fast _is the fraction of fast diffusing particles, that is, GFP free in solution. The fraction of slow diffusing particles, that is, GFP enclosed within bacterial cells, is given by 1-F_fast_. Note that these fractions are not synonymous with mole fractions, as explained in the text. The last time point for each graph represents sodium dodecyl sulfate (SDS)-induced leakage. In contrast to a sample with no additive, S1 and PMB induced leakage of *E. coli*. The control peptide negS1 induced a minor amount of leakage probably due to its high hydrophobic nature. (B) Antimicrobial peptide-induced lipopolysaccharide (LPS) release. The supernatant of fluorescent-labeled LPS molecules on bacteria was analyzed in an FCS setup. The *y*-axis represents the fluorescence photon count rate measured in kHz, that is, thousand photon counts per second. The count rate is a measure of the amount of LPS released from bacteria. In contrast to PMB (solid square), S1 (solid upward triangle) did not induce release of LPS during lysis of *E. coli*. As a positive control SDS (open diamond) was added, which disrupted the membrane therefore releasing LPS. A negative control without detergent or AMP (open downward triangle) did not cause release of LPS.

The number of particles in the confocal volume of each species is proportional to their concentration and thus changes in their relative amounts in mixtures can be determined (although the fractions determined by FCS might not correspond to mole fractions) [39]. For our purposes, we analyzed the fluctuation correlation of GFP in order to determine AMP-induced leakage from the cytosol of *E. coli*. Before addition of S1, GFP was trapped in intact bacteria and followed the diffusion times of the bacteria at approximately 0.1 to 1 second. Upon lysis, cytosolic GFP was released, showing diffusion times of only approximately 0.15 ms, that is, 10^3 ^to 10^4 ^faster than that of intact bacteria. Using FCS, we analyzed samples after addition of 500 nM AMPs or controls, at 5-minute intervals over a 1-hour period (Figure 3A).

At 500 nM S1, bacterial lysis started immediately after peptide addition. ACF curves fitted with a two-particle diffusion model showed that about 40% of GFP measured was free GFP. Bacterial lysis continued to progress as the fraction of free GFP increased steadily until a maximum of 90% was reached at about 40 min (100% corresponds to lysis with sodium dodecyl sulfate (SDS)). Addition of 500 nM polymixin B (PMB) caused bacterial lysis in a similar manner to S1 but with a slower temporal response. The control negS1 showed minor lysis activity of less than 10% on average. The hydrophobic part of negS1 is identical to S1, which could explain the minor release of GFP. In the absence of any peptide, there was no release of GFP until SDS, a membrane-disrupting detergent, was added (Figure 3A). These results suggest that S1, at a concentration of 500 nM, was highly active against *E. coli *and causes rapid lysis of the bacteria. They also suggest that S1 as well as PMB can cause membrane defects of at least 30 Å in diameter in order for GFP to leak from the bacteria [40].

To determine if S1 can disrupt the outer membrane and release LPS molecules from the lipid bilayer simultaneously, we labeled *E. coli *with a fluorescent dye on the sugar residues of LPS. After incubation with S1, the cells were pelleted and the supernatant was analyzed by FCS. Significant increase in fluorescence intensity was detected only when PMB, but not S1, was used (Figure 3B). Thus, bacterial lysis by S1 does not result in a release of LPS, in contrast to the action of PMB, as previously reported [41].

### S1 shows concentration-dependent dynamics in movement and lateral, decelerated diffusion on bacterial membranes during lysis

**Figure 4:**
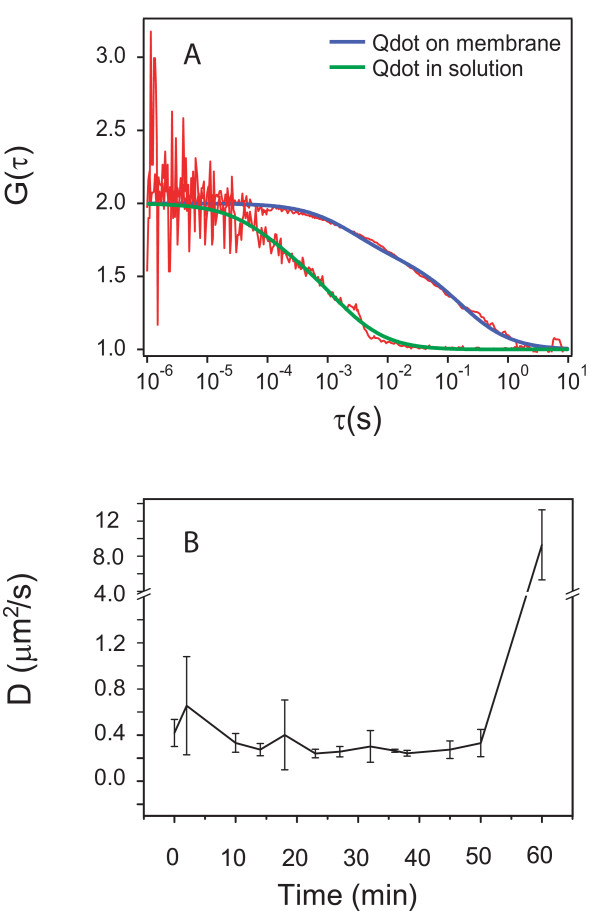
**S1-Qdot conjugate movement defined by fluorescence correlation spectroscopy**. (A) Autocorrelation function of S1-Qdot conjugate on bacterial membrane (blue curve) and free in solution (green). Concentration of S1-Qdot conjugate was 100 nM. The diffusion coefficient was between *D *= 4.96 × 10^-3 ^μm^2^/s and 0.159 × μm^2^/s. (B)Diffusion of the S1-Qdot conjugate on the membrane over time at a total concentration of S1 of 500 nM.

We employed FCS to determine diffusion coefficients of S1-Qdot on membranes of live bacteria throughout the entire lysis process. S1-Qdot was incubated with immobilized bacteria on a glass slide. Within 2 to 5 min following the addition of 100 nM S1-Qdot and 400 nM unlabeled S1, the particles diffused laterally on the membrane at *D *= 0.46 μm^2^/s, and then gradually slowed down around 11-fold to 0.04 μm^2^/s after 23 min. At the end of 1 h, the addition of SDS lysed the bacteria, releasing the AMP-Qdot into the supernatant (Figure 4A). These particles have *D *= 7.29 μm^2^/s which is comparable to that of free Qdot diffusion in solution, and approximately 18 times faster than that of bound particles (Figure 4B).

**Figure 5:**
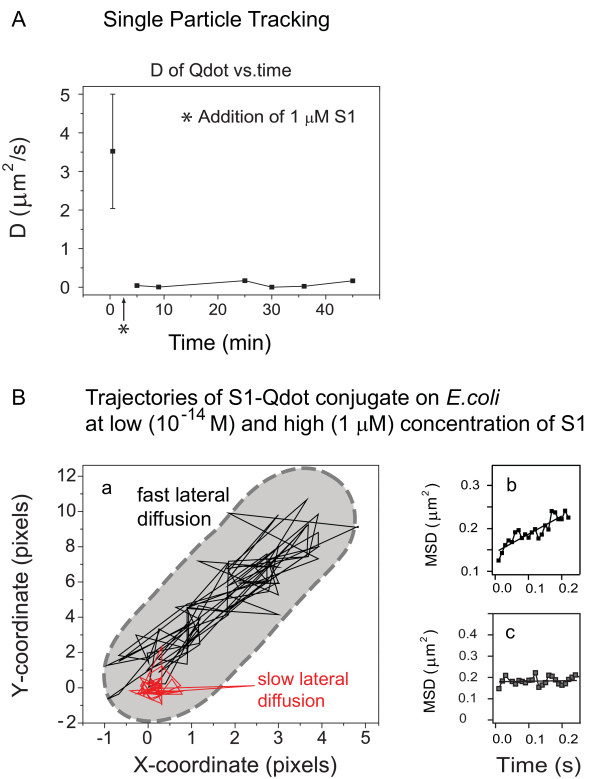
**Single particle tracking of S1-Qdot conjugate**. (A) Single particle tracking of S1-Qdot conjugate. The diffusion of the S1-Qdot particles on the membrane of *Escherichia coli *was analyzed at different time points and plotted against time. After addition of peptide to a final concentration to 1 μM, movement of the particles slowed down. (B) Movement trajectories of S1-Qdot-conjugate on *E. coli *were measured. Time on trajectories correspond to the total time during the particle movement over the bacterium. The conjugate was tracked over its *xy*-path over 100 frames. The black trajectory corresponds to the peptide movement at low concentrations of S1 (1 × 10^-14 ^M) whereas the red trajectory represents movement at 1 μM S1. Starting points were set to 0/0 (*x/y*) for both trajectories. (b) Before unlabeled peptide was added fast lateral diffusion can be observed. (c) After addition of peptide the particles show slow lateral diffusion. Data was obtained from seven independent measurements.

To confirm this observation, we characterized the peptide movement using SPT. To track single particles on bacteria we used fM (approximately 10^-15^) concentrations of the S1-Qdot. On *E. coli*, the movement of S1-Qdot conjugates was fast at low concentration of 10^-14 ^M for a Qdot:peptide ratio of 1:1. Figure 4A shows that the average D of a single S1-Qdot particle on *E. coli *was 3.52 μm^2^/s. To observe the behavior of the peptides at functionally active concentrations, we added 1 μM unlabeled S1. The movement was slowed down drastically as indicated by an approximately 100 times decrease in diffusion coefficient (from 3.52 μm^2^/s to 0.04 μm^2^/s) after 5 min, and to 6.67 × 10^-3 ^μm^2^/s after 9 min (Figure 5A). These results show that the dynamics of the particle change to a decelerated diffusion. In contrast, when 500 nM unlabeled PMB was added to fM concentrations of Qdot-PMB, the conjugated particles on *E. coli *had an average *D *value of 0.72 μm^2^/s. At a concentration of 500 nM S1, S1-Qdot showed an average D of approximately 0.06 μm^2^/s, an order of magnitude slower than the movement of PMB (data not shown).

In order to characterize the peptide movement relative to the bacterial cell, we analyzed trajectories of the particles on *E. coli*. We observed that the AMP-Qdot conjugates moved without any restriction along the bacterium (Figure 5Ba). At low peptide concentration the movement was characterized by fast lateral movements (Figure 5Bb), whereas the lateral movement slowed down at high peptide concentrations (Figure 5Bc), indicating aggregation of the peptide.

## Discussion

After bacterial invasion, innate immune response is triggered and effector molecules such as AMPs are released and bind to their targets. Upon interaction with the membrane lipids of the pathogen, S1 changes from an unstructured conformation to an α-helix. The importance of charge-charge interaction is demonstrated by results obtained with the control peptide negS1 in which cationic charges have been replaced by anionic ones. The peptide shows greatly reduced binding due to electrostatic repulsion which prevents the insertion of the hydrophobic part into the lipophilic moiety of the membrane. A possible explanation is that the initial interaction via charges is much faster than the slower insertion into the membrane by hydrophobic interaction. For further experiments we used QDot-S1 conjugates, which might give rise to concerns due to the large size of the probe relative to the peptide. However, we performed thorough testing to confirm functionality and demonstrated negligible influence of the nanoparticle in our experimental setups. Using SPT we were able to show in real-time that S1 binds to the bacterial surface and at low (fM) concentrations, travels rapidly over the membrane. As the concentration of the peptide increases, its movement decreases by a factor of 100. This indicates a multimeric association of S1 and the formation of membrane-active peptide complexes as well as the onset of lysis as shown by SPT and live bacterial lysis assays. Interestingly, S1 shows slower diffusion compared with PMB, which is known to act in a detergent-like manner [42]. This, together with the observation that S1, in contrast to PMB, does not release LPS from the bacteria, indicates that the peptides act mechanistically differently.

Binding on and destruction of the outer membrane is insufficient for killing of the bacterium since the cell wall would resist osmotic burst. Therefore an AMP has to act on both the outer and inner membranes. Using TEM on *E. coli*, we show that binding of nanogold-S1 conjugates occurs on both leaflets of both membranes, where most of the particles are localized. Only minor fractions of peptide conjugates could be found in the cytosol of the cell. A recently discussed hypothesis that AMPs might mainly be active against internal targets to kill the bacterium and not the membrane seems therefore implausible for S1 [30,43].

As seen in TEM preparations using 10 nm nanogold-peptide conjugate, S1 generates structural defects not less than 10 nm while penetrating both membranes through which the cytoplasmic components of *E. coli *leak out, leading to fast bacterial killing. However, there is no indication that S1 disrupts the membrane by acting similarly to a detergent or by forming well-defined pores. We can therefore conclude that the formation of defined pores like alamethicin is not the case for S1 [44]. Whether the formation of these structural defects is a general feature of AMPs or an S1-specific mechanism remains so far unsolved, but challenges the proposed models, pore sizes, and number of peptides per complex.

*In vivo*, we envisage that the invading bacteria are not disrupted but simply inactivated by lysis without release of LPS. The dead pathogens with S1 immobilized on their membranes are then cleared by macrophages. These observations bear a crucial advantage for the host system during infection since LPS release could trigger a severe inflammatory reaction leading to septic shock [11-13].

## Conclusion

To date, the exact mechanisms on how AMPs function on live bacteria have not been studied on a single-molecule level. The results presented here show how S1 interacts with the membranes in the steps leading to pore formation followed by bacterial death. For the first time, the entire process of bacterial killing by an AMP has been systematically monitored using a comprehensive set of end-point and real-time observations. Our results establish a temporally and spatially resolved view of the mechanism of S1, elucidating the events taking place in a biological context seen from a single-molecule perspective. Our findings suggest four successive steps of the bactericidal process of AMPs: binding, aggregation, membrane disruption, but not release of LPS, and lysis of the bacterium.

We demonstrated that the antimicrobial peptide, S1, binds to both the outer and inner membranes of GNB, leading to leakage of the cytosolic content. On the membrane, S1 shows concentration-dependent diffusive properties with reduced lateral mobility at high concentrations. During this multimeric association event, membrane defects of at least 10 nM are formed which are larger than the proposed pore sizes in the literature.

In addition to our results, the newly developed nanoparticle-conjugated AMPs represent useful tools for research in the field of antimicrobial or cell-penetrating peptides.

## Methods

### Peptides

Sushi 1 peptide (GFKLKGMARISCLPNGQWSNFPPKCIRECAMVSS) was synthesized by Genemed Synthesis Corp (San Francisco, CA, USA). S1 peptide was synthesized with and without N-terminal biotinylation. A Sushi 1 mutant named negS1 was used as negative control – the first three Lysine (K) and Arginine (R) residues were changed to Glutamate (E) – (GFELEGMAEISCLPNG QWSNFPPKCIRECAMVSS). NegS1 was produced as an N-terminal biotinylated peptide. PMB nonapeptide was purchased from Bedford Laboratories (Bedford, OH, USA). Unmodified peptides were biotinylated for conjugation by using the EZ-Link Biotinylation Kit (Pierce Biotechnology, Rockford, IL, USA) based on a protocol for N-terminal biotinylation provided by the manufacturer. Biotinylated peptides were used for conjugation with streptavidin on Qdots or nanogold. In general, the active amount of peptides was achieved by adding unmodified peptide to avoid effects of avidity. Nevertheless full activity for both the modified and unmodified peptides was confirmed by all tests.

### Transmission electron microscopy with antimicrobial peptide-nanogold conjugates

*E. coli *ATCC 25922 was grown overnight at 37°C in Mueller-Hinton broth. 1 × 10^9 ^cells/ml were washed three times with phosphate-buffered saline (PBS) and resuspended in the original volume in PBS. Nanogold particles of 10 nm size (Sigma-Aldrich) were covalently linked to streptavidin (Invitrogen), then conjugated with a 1:1 ratio of N-terminal biotinylated S1. Non-biotinylated S1 was added to a ratio of 1:5 (nanogold:peptide). 100 μl of this solution was mixed with 100 μl of *E. coli *using three different concentrations of peptide (125 nM, 500 nM and 1 μM). After varying incubation times (10, 30, 60 min) the bacteria were washed twice with 1 ml PBS each, and fixed by the addition of 2.5% glutaraldehyde and 2% para-formaldehyde, which cross-link proteins and peptides and traps carbohydrates, lipids and nucleic acids in the bacterium. Following that a 12-hour lipid fixation with 1% osmium tetroxide in PBS was performed. Subsequently a standard TEM sample protocol was followed with dehydration steps before embedding in resin [45]. After microtomy and transfer of the sample preparations onto formvar copper grids, the samples were stained by uranyl actetate (10%, 10 min) and lead citrate (1.5%, 5 min). As controls, negS1 or biotin was used together with the nanogold particles. In addition, nanogold with non-biotinylated S1 was used. The samples were observed with a Philipps CM10 TEM using a primary magnification of 42,000 to 145,000. The nanogold particles were counted to evaluate their distribution on/in the cell.

### Preparation of Qdot-labeled antimicrobial peptides

8 μM Qdot655 ITK Amino (PEG) Quantum Dots (Invitrogen) were mixed with 1 mM bis-(sulfosuccinimidyl) suberate (BS^3^, Pierce Biotechnology) dissolved in PBS (pH 7.4) in a glass vial and left at room temperature for 30 min. After buffer exchange using a desalting column (Pierce Biotechnology) the colored eluent was collected and incubated overnight at 4°C with a 1:1 ratio of streptavidin (Pierce Biotechnology). The reaction was quenched in 50 mM glycine, pH 7.5 for 2 h at room temperature. The conjugate was purified from excess of streptavidin by using ultrafiltration (100 kDa, Millipore) according to the manufacturer's instructions, exchanging the buffer to PBS, pH 7.5. Immediately before measurements, the streptavidin Qdots were incubated with biotinylated peptides for approximately~10 min under vigorous shaking.

### Fluorescence spectroscopy

Fluorescence emission spectra were recorded on a spectrofluorimeter (LS 50B, Perkin-Elmer) from 300 to 450 nm at room temperature. The excitation wavelength was set to 280 nm with both the excitation and emission slit width set to 5 nm. The spectra were baseline-corrected by subtracting the blank spectra of the corresponding lipid solutions without the peptide.

### Lipopolysaccharide neutralization assay

The LPS neutralization was quantified by using the PyroGene kit (Lonza Inc.). The principle of this assay is the activation of recombinant rFC by LPS. Upon activation the enzyme hydrolyzes a fluorogenic substrate which emits light at 440 nm when excited at 380 nm. The fluorescence was recorded at 3-min intervals. Positive (LPS only) and negative (buffer only) controls were included in each run. From triplicate measurements, the mean values with standard error and standard deviation were obtained as a function of time.

### Fluorescence microscopy

A commercial laser scanning confocal microscope (FV300, Olympus) was used to examine the staining of peptide-Qdot655 (Invitrogen) conjugates. GNB, including *Pseudomonas aeruginosa *and *E. coli*, as well as GPB (*Staphylococcus aureus*) were used. The bacteria were immobilized on 0.01% Poly-L-lysine-treated glass slides (Sigma). Then glass slides with the bacteria were washed with 1 ml PBS (16 mM phosphate buffered saline, pH 7.4) before 10 μl of 10 nM peptide-Qdot conjugate was added. Before observation, a second washing step was conducted.

### Fluorescence correlation spectroscopy instrumentation

The FCS system was built around a FV300 Olympus laser scanning confocal microscope, where an additional FCS module was coupled to the microscope. An excitation beam of 488 nm Argon laser (100 μW, Melles Griot) was reflected by an excitation dichroic mirror (560DCLP, Omega, VT) and a scanning mirror, and focused by a water immersion objective (60×, NA1.2, Olympus) into the fluorescent sample. The emission light after the confocal pinhole was focused by a lens (Achromats f = 60 mm, Linos), and separated from the excitation light by an emission filter (645AF75, Omega, VT). It was then collected on an active area of an avalanche photodiode (APD) in a single-photon-counting module (SPCM- AQR-14, Pacer Components). The transistor-to-transistor logic output signal from the APD was processed online by an autocorrelator (Flex02-01D, correlator.com) to obtain an experimental ACF curve. Curve fitting was performed using a self-written program in IgorPro (WaveMetrics). Further details on FCS theory are described in Magde *et al*. [38].

### Antimicrobial peptide-induced cell lysis assay

*E. coli *was transformed with the pEGFP vector (BD Bioscience Clontech, Palo Alto, CA, USA). This vector contains an ampicillin-resistance determinant and a GFP gene. A constitutive *lac *promoter leads to the expression of the GFP gene. We examined cell lysis ability of S1 by FCS. 20 μl of 1 μM S1 was added to 20 μl of a logarithmically growing culture of GFP-expressing *E. coli *(5 × 10^7^/ml), giving a final concentration of 500 nM S1. Then, the sample was measured using FCS over a period of 1 h at 5-min intervals. For each sample, at least eight 30-s measurements were recorded, and the mean reading was calculated. Upon cell lysis, the GFP *E. coli *release cytosolic GFP, which has a smaller diffusion time (0.15 ms) than trapped GFP, which follows the bacterial diffusion time (1 ms to 1 s). This was repeated in separate experiments with 500 nM PMB, 500 nM negS1 and no peptide (negative control) for comparison. The lysis concentration, which is indicative of the bacterial lysis ability of the AMPs, was determined. At the end of 1 h, 2.5% SDS was added to lyse the bacteria. The fraction of fast-moving particles was obtained from fitting FCS curves with a two-particle 2-D diffusion model, and plotted against time. According to the lysis concentrations and times, SPT experiments were performed. Data shown are averaged from four independent measurements.

### Lipopolysaccharide release assay

Six ml of an overnight culture of *E. coli *was washed three times with PBS. After centrifugation the bacteria were concentrated four times, and incubated with freshly prepared 10 mM NaIO_4 _at 4°C for 1 h, which oxidizes molecules such as sugars of LPS. After three more washes with PBS the bacteria were incubated with 100 μM Alexa Fluor 555 (Invitrogen) followed by 5 h at room temperature in the dark. Then bacteria were washed 10 times with PBS and diluted four times into a PBS solution containing peptides (S1, PMB) or blanks (PBS). After 10, 20, 40, 60 and 90 min, aliquots were taken and centrifuged. The supernatant was analyzed in an FCS setup and the fluorescence intensity from eight measurements were averaged and plotted against time.

### Binding of AMP-Qdot probe onto bacterial membrane

Using FCS, we measured in real-time the diffusion of 100 nM S1-Qdot conjugates upon binding to the immobilized bacteria, which have been attached to a glass slide by 0.01% poly-L-lysine. The FCS system was calibrated with 1 nM fluorescein (Invitrogen) before each experiment to ensure the proper calibration of the instrument. Then non-biotinylated S1 was added to a final concentration of 500 nM S1. At the end of 1 h, 2.5% SDS was added to lyse the bacteria. The diffusion time of conjugates moving along the membrane and when they were finally diffusing freely in solution were calculated from the ACF curves. Measurements were done in sets of eight 30-s readings.

### Single particle tracking

SPT was performed on a modified EMCCD camera (Cascade: 512B, Photometrics)-coupled inverted epifluorescence microscope (Axiovert 200 M, Carl Zeiss) in total internal reflection fluorescence mode. Laser light from a 532 nm diode-pumped solid state laser (Calypso, Cobolt, PhotoniTech) at 5 to 6 mW was used for excitation, and the emission filter used 645AF75. 10 nM S1 was incubated for 5 min in a 1:1 ratio with Qdot655, and then the mixture was serial-diluted to 1 × 10^-14 ^M. *E. coli *was immobilized onto glass slide with 0.01% poly-L-lysine, and washed in PBS. Ten microliters of 1 × 10^-14 ^M S1-Qdot was then added to the bacteria and incubated for 2 min. Unbound S1-Qdot particles were removed by washing with PBS before measurements. 1 μM unlabeled S1 was then added to the sample. Bacteria were located by differential interference contrast microscopy. Movie streams (100 frames, recording time 6 ms/frame with 5 ms exposure time and 1 ms read out time) of S1-Qdot were recorded in 80 × 80 pixel regions of interest at 5-min intervals over a 1-h period. Trajectories of S1-Qdot were tracked using Metamorph software (Universal Imaging Corp.), and mean squared displacement values were calculated from the *x*- and *y*-coordinates.

## Abbreviations

ACF: autocorrelation function; APD: avalanche photodiode; AMP: antimicrobial peptide; FCS: fluorescence correlation spectroscopy; GFP: green fluorescent protein; GNB: Gram-negative bacteria; GPB: Gram-positive bacteria; LPS: lipopolysaccharide; PBS: phosphate-buffered saline; PMB: polymixin B; Qdot: quantum dot; rFC: recombinant Factor C; SPT: single particle tracking; S1: Sushi 1; SDS: sodium dodecyl sulfate; TEM: transmission electron microscopy.

## Authors' contributions

SL developed and tested the nanoparticle probes, designed and performed transmission electron microscopy experiments and wrote the manuscript. SL and JYH designed and carried out the fluorescence correlation spectroscopy and total internal reflection fluorescence microscopy, did data acquisition and analysis and interpretation of data. JC participated in the design and coordination of the study and critically revised the manuscript for important intellectual content. BH provided cells and reviewed the manuscript. JLD and TW contributed to the conception and design of the study. All authors read and approved the final manuscript.

## Supplementary Material

Additional file 1 **Biochemical testing of S1 and the control peptide negS1**. (A) Fluorescence emission spectra of negS1 in buffer (dotted line) or in POPS (dash-dot line). For comparison S1 in buffer (solid line) and in POPS is shown (dashed line) (B) Recombinant Factor C activation assay using negS1 (open triangle) and S1 (solid circle). The activation of Factor C without lipopolysaccharide binding peptide is displayed as a solid square. The samples contained 125 nM of the peptides or no peptide. (C) Fluorescence spectroscopy of Qdot655 labeled *Escherichia coli*. After subtraction of the *E. coli *auto-fluorescence the biotin-Qdot showed a signal close to zero (dotted line). In contrast, there was a clear fluorescence peak for S1-Qdot labeled cells (solid line), negS1-Qdot show only minor fluorescence (dashed line). Settings: Excitation 488 (15 nm), Emission 600–750 (15 nm). Click here for file

Additional file 2 **Table S1**. Quantification of S1-nanoparticles counted on 20 *Escherichia coli *cells (total particle count 254) in transmission electron microscopy micrographs. Click here for file
