# Supplementary material for: Single molecule resolution of the antimicrobial action of quantum dot-labeled sushi peptide on live bacteria
Source: BMC Biol. 2009 May 11;7:22. doi: 10.1186/1741-7007-7-22 (PMC2685782; doi:10.1186/1741-7007-7-22)
Supplement: Additional file 2 — Table S1. Quantification of S1-nanoparticles counted on 20 Escherichia coli cells (total particle count 254) in transmission electron microscopy micrographs. [file 1741-7007-7-22-S2.doc]

| **Compartment** | **Particles counted** | **Distribution** |
| --- | --- | --- |
| **Outer membrane**  outer leaflet | 195 | 76.8% |
| **Inner membrane**  inner leaflet | 22 | 8.7% |
| **Periplasm**  (including outer leaflet/  inner membrane and inner leaflet/outer membrane) | 20 | 7.9% |
| **Cytoplasm** | 17 | 6.7% |
